# Towards Solving the Riddle of Forgetting in Functional Amnesia: Recent Advances and Current Opinions

**DOI:** 10.3389/fpsyg.2012.00403

**Published:** 2012-11-01

**Authors:** Angelica Staniloiu, Hans J. Markowitsch

**Affiliations:** ^1^Physiological Psychology, University of BielefeldBielefeld, Germany; ^2^Center of Excellence Cognitive Interaction Technology, University of BielefeldBielefeld, Germany; ^3^Hanse Institute for Advanced StudyDelmenhorst, Germany

**Keywords:** dissociative amnesia, psychogenic amnesia, head injury, psychosocial stress, mnestic block syndrome

## Abstract

Remembering the past is a core feature of human beings, enabling them to maintain a sense of wholeness and identity and preparing them for the demands of the future. Forgetting operates in a dynamic neural connection with remembering, allowing the elimination of unnecessary or irrelevant information overload and decreasing interference. Stress and traumatic experiences could affect this connection, resulting in memory disturbances, such as functional amnesia. An overview of clinical, epidemiological, neuropsychological, and neurobiological aspects of functional amnesia is presented, by preponderantly resorting to own data from patients with functional amnesia. Patients were investigated medically, neuropsychologically, and neuroradiologically. A detailed report of a new case is included to illustrate the challenges posed by making an accurate differential diagnosis of functional amnesia, a condition that may encroach on the boundaries between psychiatry and neurology. Several mechanisms may play a role in “forgetting” in functional amnesia, such as retrieval impairments, consolidating defects, motivated forgetting, deficits in binding and reassembling details of the past, deficits in establishing a first person autonoetic connection with personal events, and loss of information. In a substantial number of patients, we observed a synchronization abnormality between a frontal lobe system, important for autonoetic consciousness, and a temporo-amygdalar system, important for evaluation and emotions, which provides empirical support for an underlying mechanism of dissociation (a failure of integration between cognition and emotion). This observation suggests a mnestic blockade in functional amnesia that is triggered by psychological or environmental stress and is underpinned by a stress hormone mediated synchronization abnormality during retrieval between processing of affect-laden events and fact-processing.

## Introduction

Weinrich (1997), the author of “Lethe–Kunst und Kritik des Vergessens” (Lethe – Art and Critique of Forgetting) argued that human beings are by nature forgetting creatures (*animal obliviscens*) and that we frequently use the word “forgetting” to be reminded, not to forget someone or something (“Forget-me-not” has been the flower of the loving couples since the fifteenth century). As Edward Casey commented, Heidegger in “Time and Being” interpreted forgetfulness as being “more primordial than remembering” (Casey, 2000, p. 8) and Nietzsche emphasized the virtue of “active forgetfulness.”

Suggestions about the benefits of an optimum balance between forgetting and remembering can be traced back before Ribot’s time (Hacking, 1995), to the Greek mythology, where goddess *Lethe*, symbolizing forgetting complemented *Mnemosyne*, representing memory. Incidentally, exceptional mnemonical abilities were not typically identified as a blessing by their owners (Luria, 1987; Parker et al., 2006). On the contrary, similarly to the prospect that “every second of our lives recurs an infinite number of times” (Kundera, 1985, p. 5), the prospect of “running” (remembering) our entire life on a daily basis appeared as being terrifying and burdening (Parker et al., 2006). James (1984, p. 262) stated that: “If we remembered everything, we should on most occasions be as ill off as if we remembered nothing.” The disease brought by remembering everything was later on metaphorically illustrated by Borges’ character, the memorious Funes, who reaches a state where he cannot act anymore (Eco et al., 1999). On the other hand, forgetting the past is perceived as a significant threat to the identity of individuals or groups, especially in highly individualized societies. The “loss” of memory – amnesia – has been regarded as a major handicap for an individual, being often equated in the past with dementia. Media often portray societies plagued by an excessive fear of forgetting (Hacking, 1995) or being forgotten, a fear that is named athazagoraphobia.

Here we focus on discussing research data aiming to unravel the “mystery” of forgetting in so-called functional amnesia. After defining functional amnesia, we briefly outline the memory systems and their main neural correlates. We then review clinical, epidemiological, neuropsychological, and neuroimaging findings of functional amnesia and discuss the pathophysiological mechanisms of “forgetting” in functional memory “loss,” by preponderantly resorting to own patient research data. We emphasize that both prototypical and cases that radiate from the prototype (“radial classes,” Hacking, 1995) are essential for the understanding of this condition and discuss the diagnostic challenges posed by the radial cases.

### What is functional amnesia?

The meaning of the term functional amnesia has undergone changes over time. Initially seen as the antipode of so-called “organic” amnesia, the use of the term functional amnesia shifted to designate amnesic disorders that occur without evidence of significant brain damage as detected by conventional structural brain imaging techniques and have an unsure etiology. When brain damage is present, the extent and/or nature of amnesia do not match the locus and/or severity of the brain lesion (Piolino et al., 2005). Although several authors still use the terms psychogenic, dissociative or functional amnesia interchangeably, implicitly acknowledging that a number of functional amnesias have a psychological basis (Kritchevsky et al., 2004), some differences among the theoretical scaffolding of these terminologies exist. The term dissociative amnesia defines a form of psychogenic amnesia underlain by the psychological mechanism of dissociation (Janet, 1907, p. 23). It belongs to the dissociative disorders in DSM-IV-TR (2000), which are characterized by subjectively perceived disturbances of the integrated organization of memory, perception, consciousness, identity, or emotion, and are regarded as being causally bound to psychological trauma or stress (Spiegel et al., 2011). In contrast to the term dissociative amnesia, which is theoretically charged *a priori*, the term psychogenic amnesia refers to amnesic disorders that are etiologically linked to a larger variety of psychological mechanisms (McKay and Kopelman, 2009). The concept of functional amnesia (Schultz, 1924; Lundholm, 1932) was suggested by De Renzu et al. (1997, p. 788) as a “more suitable term to classify patients whose memory disorders cannot be traced back to organic or psychological causes.” Several cases of functional amnesia were found to occur on a background of psychological stress or trauma, alone or in combination with a co-occurring physical insult. This co-occurring physical insult is usually of mild severity (such as a mild traumatic brain injury or electrocuting accident; e.g., Tramoni et al., 2009), but more severe physical insults were described as well (Fast and Fujiwara, 2001; Pommerenke et al., 2012). In some case reports of functional amnesia, a clear-cut psychological etiological mechanism could not be identified (De Renzu et al., 1997; Fujiwara et al., 2008). The lack of identifiable psychological factors in some cases of functional amnesia may be accounted for by the retrograde amnesia itself, by impaired emotional processing, or the so-called incubation effect of life adversity (Post et al., 1995; Lupien et al., 2009). As Kopelman (2000) pointed out, the identification of precipitating psychological factors in cases of amnesia is not always straightforward (Markowitsch et al., 1993). It may require a perseverant archeological work to get to the well of the illness.

A minority of cases of so-labeled functional amnesias may represent early stages of particular neurodegenerative forms of amnesia with early age of onset, where the morphological changes initially might escape capturing by conventional structural brain investigations, but may declare themselves afterward (Ladowsky-Brooks and Fischer, 2003). There are reports in the literature of conditions that had initially been deemed as functional or hysterical, but subsequently re-classified as due to a general medical condition (Slater and Glithero, 1965). Stone (2009, p. 186) however remarked that “‘misdiagnosis’ for ‘conversion symptoms’ in studies since 1970s has on average been around only 4% at 5 years.” He stated further: “This is the same as for other neurological and psychiatric conditions such as multiple sclerosis and schizophrenia” (p. 186). Albeit occurrences of misdiagnosis may be less frequent nowadays (Stone, 2009), they nevertheless bring us to the critical points that had been raised with respect to using the term functional amnesia (Kanaan et al., 2012). McKay and Kopelman (2009, p. 152) argued that the “term ‘functional’ amnesia has the problem that the amnesia could in many respects be considered dysfunctional.” Furthermore, the assertion that the term functional “in its proper sense means that the lesion affects function and not substrate” (De Renzu et al., 1997, p. 788) is nowadays challenged by findings of studies that combined functional and newer structural neuroimaging methods (Tramoni et al., 2009). Since the advent of functional imaging, research data have increasingly been providing evidence in functional amnesia of functional and metabolic changes in brain areas that are agreed upon to exert a crucial role in memory processes (Markowitsch, 1999a; Brand et al., 2009; Staniloiu and Markowitsch, 2010b; Thomas-Antérion et al., 2010). Additionally, the recent broader use of imaging techniques such as diffusion tensor imaging or magnetization transfer ratio measurements started to provide evidence in this condition of subtle micro-structural changes of fiber tracts involved in conscious mnemonic processing (e.g., Tramoni et al., 2009), questioning therefore the above understanding of the construct “functional” put forth by De Renzu et al. (1997). Another challenge to this concept comes from more recent data showing that, in contrast to old views of functional amnesia as a reversible condition, in a substantial number of patients with functional amnesia the (episodic-autobiographical) memory impairments followed a chronic, unremitting course (Kritchevsky et al., 2004; Fujiwara et al., 2008). In some cases there may even be cognitive deterioration over time (Kessler et al., 1997).

Despite its imperfections, the construct functional amnesia seems better suited for capturing this condition than other terminologies (Stone, 2009). It furthermore provides a common “intellectual” framework for studying psychiatric and neurological diseases (Markowitsch, 1999b; Stone, 2009; Kanaan et al., 2012). As Markowitsch (1999a, p. 335) stated “A common framework for psychiatry and neurosciences emphasized 115 [years] ago by Meynert who attempted to explain all psychic functions by anatomy and physiology […], is nevertheless a central aim for … the next century […].”

### Memory systems

Understanding various types of functional amnesia might be aided by a brief overview of the current main classifications of the memory systems and processes. Memory is not a unity, but is divided along the time and content dimensions. Along the time axis, short-term memory has been limited to a few bits (4–7) and a time range of seconds to minutes (Miller, 1956; Cowan, 2000). Baddeley extended the concept of short-term memory by adding that of “working memory” (Baddeley and Hitch, 1974; Baddeley, 2000). Working memory involves the online-holding not only of new information, but also the retrieval of components of old, already stored information (Aben et al., 2012).

Another important time-bound categorization involves the distinction between old and new memories, and anterograde and retrograde amnesia, respectively. The term anterograde amnesia was reportedly advanced by Charcot to account for the “pathological forgetting” of events that happened after the traumatic event (Janet et al., 2001). Following Etienne Eugéne Azam (1822–1899), Charcot reportedly used the construct “retrograde amnesia” (Janet et al., 2001). With respect to the classification of long-term memory according to content, already on the beginning twentieth century, several authors suggested the existence of various forms of memory (Semon, 1904; Ziehen, 1908; Schneider, 1912, 1928). The work of Tulving (1972) – in human research domain, and that of Mishkin and Petri (1984) – in animal research, promoted divisions between the episodic and semantic memory (Tulving, 1972, 1983) or the memory and habit system (Mishkin and Petri, 1984). These divisions were followed by more refined categorizations, encompassing procedural, and priming memory system (Squire et al., 1993; Tulving, 1995). Five long-term memory systems were ultimately proposed to exist by Tulving and Markowitsch [“procedural memory,” “priming,” “perceptual memory,” “semantic memory,” “episodic(-autobiographical) memory”; cf. e.g., Markowitsch, 2003; Tulving, 2005], which are considered to build up ontogenetically and phylogenetically onto each other (see also Nelson and Fivush, 2004).

**Figure 1:**
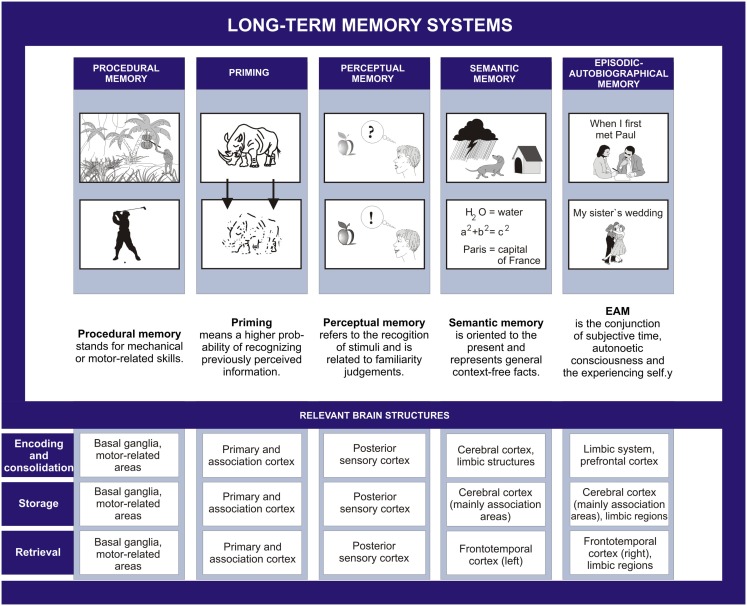
**The five long-term memory systems**.

These systems have distinct or partly distinct neural substrates and different degrees of susceptibility to environmental insults (Figure 1). They are assumed to be accompanied by different levels of consciousness. The first two memory systems (priming and procedural memory) are devoid of the need for consciousness (“anoetic”). [The terms autonoetic (“self-conscious,” or “self-aware”), noetic (“aware”), and anoetic (“not-aware”) were introduced and elaborated on by Tulving, 1995]. Perceptual memory system, which is the newest addition to the long-term memory systems, acts consciously (“noetically”), but on a presemantic level, and relies on familiarity judgments. It enables the identification of an apple, no matter what color it has or whether it is already half eaten or not; it also allows distinguishing an apple from a pear on the presemantic level. The mnemonic performance in the perceptual memory system remains however insufficiently explored experimentally, perhaps due to a lack of consensus among the researchers about what perceptual memory means (Eustache and Desgranges, 2008).

Semantic memory comprises general semantic knowledge (school and world knowledge) and autobiographical-semantic knowledge (knowledge of name, date of birth, or self-traits). The episodic memory is presently defined as the conjunction of subjective time, autonoetic consciousness, and the experiencing self (Tulving, 2005) and the episodic memory system is considered to be equivalent to the episodic-autobiographical memory system (Markowitsch and Staniloiu, 2012a,b). While semantic memory is accompanied by noetic consciousness, episodic-autographical memory features a superior level of consciousness (autonoetic consciousness). Autonoetic consciousness entails a “sense of self in time and the ability to relive subjective experiences from the encoding context by mentally traveling back in time” (Lemogne et al., 2006, p. 260). Mental time traveling through subjective time from present to both past and future is according to Piolino et al. (2009) one of the last features of episodic-autographical memory that becomes fully functional, but the first one to be afflicted by aging and amnesic conditions (see also Markowitsch and Staniloiu, 2012a). For the present article, especially the episodic-autobiographical memory system is of importance, as this is consistently and often severely affected in patients with functional amnesia.

Episodic-autographical memory is considered to develop the latest phylogenetically, being arguably uniquely human (Clayton and Dickinson, 1998; Tulving, 2005). Ontogenetically, episodic-autographical memory represents the last acquisition (Nelson and Fivush, 2004; Tulving, 2005; Piolino et al., 2007), emerging in concert with other cognitive abilities (Nelson and Fivush, 2004; Wang, 2008; Picard et al., 2009). The earliest childhood episodic-autographical memory typically dates to around age 3.5 years, but individual, cultural, and gender differences exist (Wang, 2001; Harpaz-Rotem and Hirst, 2005; Davis et al., 2008). Developmental changes of episodic-autographical memory and to some degree of autobiographical-semantic memory may however extend beyond childhood into early adolescent years (Picard et al., 2009; Willoughby et al., 2012).

### The neuroanatomical bases of memory

The present theoretical approach to memory processing emphasizes that both specialization and integration are features of the human brain (Paus, 2010). It is assumed that information enters the brain via the sensory organs and is then further processed according to the kind of information and the process selected or triggered. This implies that subconsciously processed information is directly led to unimodal neocortical structures (priming), or engages the basal ganglia, premotor, and other motor-related areas (procedural learning). Consciously processed information engages more widespread networks which are still largely neocortical for perceptual learning, but include limbic regions for the semantic memory and episodic-autographical memory systems. For the latter two memory systems incoming information first enters limbic structures where its biological and social relevance is extracted, the information is compared with already existing related information and later bound to and integrated with these (synchronization; Axmacher et al., 2006). Further consolidation occurs during sleep (Stickgold and Walker, 2005). Incidentally, a pronounced number of patients with functional amnesia report sleep disturbances (Loewenstein, 1991). Modest correlations between sleep disturbances and dissociative experiences were found, though their meaning is incompletely understood (Lynn et al., 2012). A labile sleep-wake cycle might lead to dream-like experiences, via intrusions of sleep-related material into the wakefulness, which might in turn promote dissociative experiences. Furthermore, a labile sleep-wake cycle might impact on cognitive processing, such as mnemonic processing and attentional control (Lynn et al., 2012).

The process of memory consolidation may extend to years (Haist et al., 2001); this might hamper the accurate identification of the mechanisms (retrieval versus consolidating deficit) underlying certain amnesic conditions (Miller and Sweatt, 2006). Widespread nets within the cerebral cortex are seen as pivotal for storage of memories (Markowitsch, 1985; Mesulam, 1998; for a description of the nets, see the next paragraph). Storage is however never final, as new information or the retrieval of already existing one leads to re-consolidation and new storage in the context of the last re-consolidation (Wood, 2003; Fulton, 2006; Nader and Hardt, 2009; Nadel et al., 2012). This constitutes the basis for pharmacological studies that aim to weaken traumatic, disturbing memories by interfering with their assumed post-retrieval re-consolidation (Brunet et al., 2008).

Retrieving facts and events requires the engagement of three intimately interacting networks, namely activating brain stem structures comprising portions of the reticular activating system, the main information of the respective fact or event containing neocortical network plus – especially for the events or episodes – a limbic network providing the emotional flavor (Anderson et al., 2006). According to Tulving’s SPI model (serial, parallel, independent = SPI), information is encoded serially, may be stored in parallel in different memory systems and can be retrieved independently of the system in which encoding occurred. The SPI model is of importance for patients with functional amnesia. As Spiegel et al. (2011) remarked, the preserved ability to retrieve information from other memory systems than the episodic-autobiographical one might have led researchers or clinicians to wrongly conclude that the person’s amnesia was feigned.

Data from single and group studies of neurological patients emphasize the importance of regions of the limbic system for encoding episodic-autographical and, to a lesser extent, semantic memories (see Markowitsch and Staniloiu, 2012a). The most investigated region of the limbic system is the hippocampal formation, due to the seminal writings on patient Henry G. Molaison by Brenda Milner (see Corkin, 2002; Squire, 2009). After undergoing neurosurgery for pharmacologically intractable seizures, the premorbidly cognitively intact young man Henry Molaison became severely anterogradely and in part retrogradely amnesic; longitudinal follow-up indicated that his cognitive performance might have further deteriorated over time compared to individuals of his age. His main impairment after brain surgery was his inability to consciously acquire factual and episodic information and events for long-term storage (cf. Figure 1 of Squire, 2009); in addition he showed – when tested several years later – deficits in the conscious retrieval of events. Several researchers concluded that the region within the medial temporal lobe responsible for the severe memory impairment in the episodic-autobiographical domain was the hippocampus proper (e.g., Vargha-Khadem et al., 1997), while surrounding structures – damaged in Henry Molaison as well – might be necessary for semantic memory processing.

**Figure 2:**
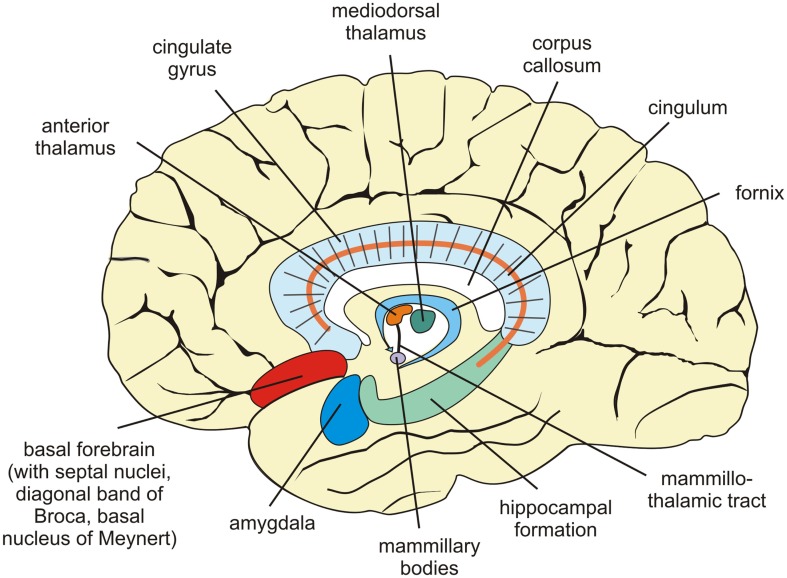
**Sagittal view of the human brain showing structures centrally implicated in information processing, especially with respect to the transfer of newly acquired memories for long-term storage**.

Other regions within the limbic system are important bottleneck structures for episodic-autographical memory encoding (Brand and Markowitsch, 2003; Markowitsch, 2008; Markowitsch and Staniloiu, 2012a), such as medial and anterior diencephalon, the amygdala, and the basal forebrain (septal nuclei, basal nucleus of Meynert, diagonal band of Broca; Figure 2). Bilateral damage to the medial (and to a certain degree to the anterior) diencephalic structures leads to severe anterograde amnesia. As this region contains a number of fibers (mammillothalamic tract, internal medullary lamina), some amnesic conditions may be viewed as a “disconnection syndrome” due to the disruption of connections between distant brain structures (Markowitsch, 1988).

Within the hypothalamus, the mammillary bodies were implicated in conscious memory processing a long time ago (Markowitsch, 1992). This was largely due to their degeneration in patients with Korsakoff’s syndrome (Markowitsch, 2010; Markowitsch and Staniloiu, 2012a). The Korsakoff’s syndrome is the prototypical example of diencephalic amnesia and is characterized by both anterograde and retrograde impairments of mainly episodic-autographical memory, but sometimes semantic memory as well, as well as confabulations and disorientation with respect to time and place. The syndrome is typically described in patients with chronic alcohol abuse and vitamin B_1_ (thiamine) deficiency or various medical conditions with severe thiamine deficiency (cf. Markowitsch, 2010).

As the two amygdaloid nuclei are located widely apart, they are rarely damaged exclusively and bilaterally. An exception is the Urbach–Wiethe disease, a genetic condition that can lead to a selective calcification of both amygdala (Markowitsch et al., 1994). As a consequence of bilateral damage of amygdala-structures, patients with Urbach–Wiethe disease may suffer problems with the processing of emotionally laden episodic-autobiographical information (Cahill et al., 1995; Siebert et al., 2003). The amygdala contributes to encoding, consolidating, and retrieval of emotional personal events. A working tandem may exist between the amygdala and septal nuclei (von Cramon and Markowitsch, 2000). Activity of a normal septum dampens emotional processing, while activity of a non-damaged amygdala enhances it.

As outlined above, the limbic system structures are engaged in a complementary, but closely interwoven way in acquiring episodic-autographical information, while neocortical and in part limbic areas represent the major storage places. The combined activation of right hemispheric fronto-temporal regions serves as trigger stations for retrieving stored episodic-autographical events (Brand and Markowitsch, 2006; Markowitsch and Staniloiu, 2012a). The corresponding regional complex in the left hemisphere seems to trigger the retrieval of old semantic information (Markowitsch and Staniloiu, 2012a). Frontal regions as well as parietal areas have been attributed roles in mental time traveling and subjective time coding (Nyberg et al., 2010).

The fiber system, interconnecting the fronto-temporal regions is the uncinate fascicle. The right uncinate fascicle contains 33% more fibers and is 27% larger than the left one (Highley et al., 2002). It was ascribed functions in episodic-autobiographical memory and emotional processing (Markowitsch, 1995b).

Retrograde amnesia for personal events might arise from a synchronization abnormality during retrieval between processing of affectively laden personal events (that is assumed to preferentially and preponderantly engage the right hemisphere) and fact-based processing (that is considered to preferentially and preponderantly engage the left hemisphere; Fink et al., 1996; Markowitsch et al., 2000b). It might be due to a neurological event that leads to damage of right fronto-temporal connections (such as the right uncinate fascicle; Levine et al., 1998, 2009; LaBar and Cabeza, 2006; Fouquet et al., 2009) or a psychological stress-induced right fronto-temporal disconnectivity (Reinhold et al., 2006; Reinhold and Markowitsch, 2007, 2009; Brand et al., 2009). Right fronto-temporal disconnections have also been described in cases of functional amnesia triggered by a mixture of psychological and physical factors (Piolino et al., 2005; Tramoni et al., 2009).

### Functional amnesia and its types

The memory impairment in functional amnesia is usually of a retrograde nature, but might at times be anterograde as well. Although impaired recall of autobiographical events is the most prominent symptom in psychogenic amnesia, deficits in retrieving personal facts, and general semantic (non-personal) knowledge can also occur (Barbarotto et al., 1996; Kritchevsky et al., 2004; Fujiwara et al., 2008). In addition, variable degrees of anterograde memory deficits (as detected by *standard* anterograde memory tests; Markowitsch and Staniloiu, 2012a) can accompany the retrograde amnesia. However, in most patients with functional amnesia, anterograde mnemonic processing was reported to be preserved to a large extent (De Renzu et al., 1997; Glisky et al., 2004; Brand et al., 2009).

Often, anterograde amnesia occurs initially and it then resolves or becomes mild. The delay between the onset of functional amnesia and the time of neuropsychological study may lead to an underestimation of the anterograde memory deficit that could have been part of the initial picture of the functional amnesic condition (Coons and Milstein, 1992; Kopelman, 2000). In comparison to functional retrograde amnesia, cases of functional anterograde amnesia with preserved retrograde episodic-autobiographical memory (Kumar et al., 2007; Smith et al., 2010; Staniloiu et al., 2010a), or cases of functional amnesia with anterograde memory impairments out of proportion in comparison to the retrograde memory impairments, have less frequently been reported or acknowledged (reviewed in Markowitsch, 1992). In contrast to the DSM-IV-TR (2000) description that popularizes the view of dissociative amnesia as being prototypically retrograde, Janet described several patients with anterograde amnesia that involved a mechanism of dissociation (“psychological disintegration”). He observed that in these cases “instead of losing engrams that they have acquired, patients do not acquire any engram” (Janet et al., 2001, p. 485). One of these patients was Mrs. D, a 34-year-old woman who was studied both by Souques (1892) and Janet (1892) at the invitation of Charcot. After suffering an emotional traumatic event, Mrs. D developed retrograde amnesia spanning a period of almost 4 months prior to the incident as well as profound anterograde amnesia. According to Janet, her speech and reasoning were intact, she recalled everything she had learned before, but was forgetting all new information presented to her after 2 or 3 min (Janet et al., 2001). Several historical cases that had been labeled as Korsakoff’s syndrome would nowadays better fit criteria for a diagnosis of functional amnesia (Markowitsch, 1992).

Functional amnesia may affect memories from across the whole life-span or just those from a specific time period or with a specific content. For instance, some patients “forget” events which happened within a clear time window, for example within the last 6 years, or within the last 13 years before the critical incident. With respect to content, the amnesia may affect all autobiographical memories, or be restricted to specific autobiographical material.

Retrograde psychogenic amnesia might be accompanied by suddenly leaving the customary environment and compromised knowledge about personal identity. This variant was named dissociative or psychogenic fugue (Markowitsch et al., 1997). Dissociative amnesia or fugue may co-occur with other dissociative or somatoform disorders, such as conversion disorder or Ganser syndrome. The Ganser syndrome is currently considered a Dissociative Disorder Not Otherwise Specified in DSM-IV-TR (2000), where it is solely defined by giving approximate answers to questions (*vorbeireden*). Ganser’s (1898, 1904) original description of the syndrome however encompassed a hysterical semitrance, a tendency to give approximate answers, amnesia, and hallucinations. The Ganser syndrome had initially been described in young men with a forensic background. Later on the syndrome was reported in non-forensic contexts and women and children as well (Nardi and Di Scipio, 1977), with a substantial number of cases being found in migrants or ethnic minorities (for a review, see Staniloiu et al., 2009). In Ganser syndrome comorbidities are common and epidemiological data are insufficient (Dalfen and Feinstein, 2000). As Dwyer and Reid (2004, p. 473) stated, all in all “Ganser’s syndrome remains an enigma, but despite its rarity it should not be forgotten, for it serves to highlight the limitations of our understanding of the disordered mind.”

### The epidemiology of functional amnesia

Little is known about the epidemiology of functional amnesia *per se*. This is partly due to the fact that “functional amnesia” currently designates no diagnostic entity in international classifications of diseases (ICD-10, 1992; DSM-IV-TR, 2000). Although its prevalence or incidence cannot be ascertained, there are hints that its frequency is underestimated in the research field (Brandt and van Gorp, 2006). Brandt and van Gorp (2006, p. 332) asserted that “all the dissociative disorders are extremely rare.” Some authors concluded that certain dissociative disorders simply do not exist in their countries (Takahashi, 1991; Fujii et al., 1998) and others viewed their existence as being the product of cultural contamination (Xiao et al., 2006; Pope et al., 2007). Coons and Milstein (1992) stated that the incidence of psychogenic amnesia among their adult service patients was 1.8%. Furthermore, they commented that the condition may be overlooked when subtler presentations exist or when other primary psychiatric conditions are superimposed. In a recent review article, Spiegel et al. (2011) noted that the reported prevalence values for dissociative amnesia range between 1.8 and 7.3%. Johnson et al. (2006) reported for dissociative amnesia a 12-month-prevalence of 1.8% in a USA community of 658 adults who were assessed with psychiatric interview.

The sex ratio in dissociative amnesia was reported to be roughly balanced (Maldonado and Spiegel, 2008). Some studies however described a higher frequency of psychogenic amnesia in women (Coons and Milstein, 1992), while others in men (Kanzer, 1939; Fujiwara, 2004; Kritchevsky et al., 2004).

Most of the cases of psychogenic amnesia are diagnosed in the third or fourth decade (Schacter et al., 1982; Coons and Milstein, 1992; Fujiwara, 2004). In the sample of Coons and Milstein the age ranged from 17 to 51 years. The mean educational level was 12.7 years; the years of education, however ranged from 9 to 18 years (Coons and Milstein, 1992). These findings are consistent with results from other studies (Fujiwara, 2004; Kritchevsky et al., 2004; Brand et al., 2009). With respect to marital and employment status, the findings vary among studies (Coons and Milstein, 1992; Fujiwara, 2004). Coons and Milstein (1992) found that 40% of their sample had recurrent episodes of psychogenic amnesia. Abeles and Schilder (1935) reported a recurrent rate of 24%. Coons and Milstein (1992) attributed the elevated rate of recurrence in their sample to the nature of their assessment setting (a tertiary care unit).

### The neuropsychology of functional amnesia

The neuropsychological examination of patients with functional amnesia reveals several characteristics. Patients with functional amnesia often present with impairments of episodic-autobiographical memory, which are of retrograde nature. In comparison to the symptom of amnesia that may occur in post-traumatic stress disorder, the memory impairment in functional amnesia is usually more extensive, encompassing larger periods (Spiegel et al., 2011). With respect to semantic knowledge, autobiographical-semantic knowledge may initially be impaired, but then it is relatively quickly re-learned. Old general world knowledge is typically preserved, but in some cases may be affected in variable degrees (Kopelman, 2000; Brand et al., 2009). Perceptual memory has not been formally studied in patients with dissociative or functional amnesia. However, there are reports of variable impairments of familiarity judgments in patients with functional amnesia (Kanzer, 1939; Pommerenke et al., 2012); one could therefore speculate that at least in a subset of patients the mnemonic processing in this system may transiently or partly be affected as well. A limited number of studies of patients with functional amnesia provided objective evidence for impairments of previously learned procedural skills or for deficits in acquiring new procedural skills. Given that many patients quickly relearn their skills, their initial complaints may reflect an impairment in the “procedural discourse” (Duff et al., 2008), due to impairments in the semantic memory domain (De Renzu et al., 1997). In other cases the “loss” of skills may just represent difficulties with initiating or carrying out a task, either due to emotional-motivational factors or executive dysfunctions (Janet, 1907; Fujiwara, 2004; Smith et al., 2010). The capacity for imagining future personal events is often affected in patients with functional amnesia (Markowitsch and Staniloiu, 2011a). A deficient performance on various prospective memory tasks may be accounted for by impairments in episodic-autobiographical mnemonic processing, semantic mnemonic processing, and/or executive dysfunctions (Markowitsch and Staniloiu, 2012a). Executive functions have been reported to be affected in a substantial number of patients with functional amnesia (Fujiwara et al., 2008; Brand et al., 2009). Already Janet described in patients with hysteria an incapacity of “beginning” or “stopping” an “act” as well as problems with making decisions (Janet, 1907). The executive dysfunctions in patients with functional amnesia may compound the impairments in conscious memory retrieval. They may also contribute to abnormalities in social information processing (social cognition and regulation; Bull et al., 2008). Various impairments of self-referential processing have been reported in patients with functional amnesia. A profound loss of personal identity may accompany dense retrograde forms of functional amnesia. Anomalous self-face processing has been described in several cases (Markowitsch and Staniloiu, 2011a; Pommerenke et al., 2012) and linked to a right hemisphere dysfunction (Abe et al., 2007). Impairments in self projection in the form of an impaired ability to imagine future personal events (Addis et al., 2009) are often reported in patients with functional amnesia. The ability for self-reflection may be reduced due to a “retraction” or “narrowness” of the “field of consciousness” (as suggested by Janet, see Dorahy and van der Hart, 2007) or a shift from the autonoetic consciousness to a noetic type of consciousness. “Autonoetic consciousness is characterized by a superior ability to reflect upon oneself and to distinguish oneself from the social and biological environment” (Markowitsch and Staniloiu, 2011a, p. 719). The lack of autonoetic consciousness in functional amnesia was also emphasized by Sullivan (1972); he stated: “In the fugue, the self is involved, but the consciousness is not of the type to which I would refer as fully developed. Paradoxically, there may be conscious involvement of the self without the degree of integration to which we may apply the term *self-consciousness* in its usual sense” (p. 288).

Impairments in self-regulation can reflect a malfunctioning of the right inferolateral prefrontal cortex. This malfunction can occur not only in patients with dissociative amnesia, but was also reported in patients with bulimia nervosa (Marsh et al., 2009). Incidentally, bulimia nervosa is one of the possible comorbidities of dissociative amnesia (Maldonado and Spiegel, 2008). Obesity and binge eating were also listed as possible comorbidities of psychogenic amnesia by Coons and Milstein (1992). Interestingly, after the onset of functional amnesia, changes in eating preferences have been reported, which may have various explanations (Fujiwara, 2004; Fujiwara et al., 2008). One possible explanation is that they reflect a change in personality dimensions (Enzi et al., 2009). Another (unexplored) explanation is that they may be related to changes in the sense of smell. After mild traumatic brain injuries, changes in olfaction are common, although patients may not be aware of them. A substantial proportion of the changes in taste occur in fact as a result of changes in olfaction (Haxel et al., 2008).

Another possible explanation is that several structures involved in processing within the episodic-autobiographical memory system (such as amygdala, parts of basal forebrain, ventro-medial prefrontal cortex) have also been reported to be engaged in reward-related processing (e.g., Nieuwenhuys et al., 2008). There is currently increasing data about the interaction between the reward system and psychological stress (Admon et al., 2012). Not only changes in eating preferences have been reported in patients after the onset of functional amnesia, but also changes in smoking and drinking habits (Kritchevsky et al., 2004; Fujiwara et al., 2008; Thomas-Antérion et al., 2010). One patient with a variant of dissociative amnesia (dissociative fugue) lost his allergies and asthma after the onset of retrograde amnesia (Markowitsch et al., 1997). This finding is interesting, in the light of data showing that immune functions can be changed or modulated by associative learning processes (Vits et al., 2011).

Conversion or depersonalization symptoms may accompany certain variants of functional amnesia and augment the sense of identity loss (Arzy et al., 2011). These conditions may reflect, apart from a fronto-limbic disconnection (Black et al., 2004), a malfunctioning in parietal areas important for body schema, mental time traveling, and time processing (Hoff and Pötzl, 1938; Metzinger, 2008; Nyberg et al., 2010). Various impairments of time processing have been reported to occur in patients with functional amnesia (Markowitsch, 2005). Social information processing (social perception, cognition, and regulation) may be impaired in patients with functional amnesia (Adolphs, 2010). The capacities for theory of mind, empathy, and social and moral judgments may be affected (Fujiwara et al., 2008; Reinhold and Markowitsch, 2009; Croft et al., 2011). With respect to social regulation, emotional dysregulation, and reduced self-reflection may occur (Markowitsch et al., 1997).

Abnormalities of emotional processing in patients with functional amnesia are not surprising. Although the most recent definition of episodic memory does not make any reference to emotion, several authors have emphasized the intimate connection of episodic-autobiographical memory with emotion (Markowitsch and Staniloiu, 2011b). Comorbidities with affective disorders (major depressive disorders) or subclinical depressive symptoms are common in patients with dissociative amnesia and they may have as foundation a shared neurobiological mechanism (Simeon et al., 2007; Staniloiu et al., 2009).

According to international nosological criteria, the memory impairment in amnes(t)ic disorders should not be due to dementia or occur exclusively during delirium. In ICD-10 it is often specified that in amnesic disorder the “immediate” recall should be preserved. Functional amnesia might pose a challenge to the above mentioned diagnostic criteria. Sometimes patients with functional amnesia present with a global intellectual deterioration, suggestive of a dementic picture (Spiegel et al., 2011). While in some cases, this ultimately proves to be a pseudodementia picture that remits, in other cases the cognitive decline may persist or even worsen over time (Markowitsch, 1992). Alterations of consciousness may at times be profound, such as in Ganser syndrome or in amnesia accompanying dissociative possession trance (Markowitsch, 1992; Staniloiu et al., 2009; During et al., 2011). These alterations have been named hysterical semitrance or stupor or pseudo delirium (DSM-IV-TR, 2000).

Impairments of short-term memory may also occur in patients with functional or dissociative amnesia (Janet, 1907; Fujiwara, 2004; Fujiwara et al., 2008) and they partly may be due to the presence of comorbidities. Apart from comorbidities with affective disorders, the co-occurrence of eating disorders (bulimia nervosa), somatoform disorders, conversion disorders, alcohol misuse, anxiety disorders, and personality disorders (narcissistic, borderline, antisocial) have also been described. While the generalizability of these findings awaits confirmation from well-designed epidemiological studies, nevertheless the proponents for DSM-V suggested a change in the criteria for dissociative amnesia to allow room for the presence of comorbidity (Spiegel et al., 2011). Long lasting personality changes have been reported in the context of the onset of functional amnesia (Kritchevsky et al., 2004). This shift in personality may modify enduring patterns of inner experience and behavior that pertain to both affectivity and cognition (DSM-IV-TR, 2000).

A lack of concern about the current situation has been described in substantial number of patients with functional amnesia and has been termed “la belle indifférence” by Janet (1907); its psychological and neurobiological mechanisms are not fully understood yet (Stone et al., 2006). Furthermore, “la belle indifférence” neither was reported to occur in all cases of functional amnesia, nor was described to happen exclusively in amnesia of functional (psychogenic) origin (Wilson et al., 1950; Fujiwara, 2004).

### The course of functional amnesia

Functional amnesias can be classified into acute and chronic, respectively. Amnesias persisting over a month period have been deemed chronic. This duration cut-off for chronicity may stem from older studies, which showed that a high percentage of psychogenic amnesias resolved within a month from their onset (Abeles and Schilder, 1935; Kanzer, 1939; Loewenstein, 1991). For the last two decades, several authors have reported that in a substantial number of patients with functional or psychogenic amnesia the memory impairment follows a chronic course (Coons and Milstein, 1992; Kritchevsky et al., 2004; Fujiwara et al., 2008; Thomas-Antérion et al., 2010). The discrepancy between older and newer studies with respect to the course of amnesia may be accounted for by several factors, such as the referral bias, the employment of increasingly sophisticated neuropsychological methods for the investigation of memory impairment and the changes, which occurred in the conceptualizations of episodic-autobiographical memory (Coons and Milstein, 1992; Kritchevsky et al., 2004; Tulving, 2005; Spiegel et al., 2011; Barnabe et al., 2012).

### Mechanisms of forgetting in functional amnesia

#### The psychological stress model: stress and episodic-autobiographical memory retrieval blockade

Albeit some authors still dispute their legitimacy, regarding them exclusively as a culture-bound syndrome that flourished in the 1800s (Pope et al., 2007), dissociative (psychogenic) amnesic disorders have been linked to psychological trauma or stress in a variety of cultures (Thom and Fenton, 1920; Kiersch, 1962; Spiegel and Cardena, 1991; Draijer and Langeland, 1999; Xiao et al., 2006; Jones et al., 2007; Seligman and Kirmayer, 2008). As Goldsmith et al. (2009) remarked, Pliny the Elder (23–79 A.D.) already had talked about “fright” as being one of the causes of partial or total memory “loss.”

In line with these views, Markowitsch developed a model of functional amnesia that postulates that the memory impairment in the episodic-autobiographical domain in functional retrograde amnesia preponderantly reflects a stress hormone mediated memory blockade, underpinned by right hemispheric synchronization abnormalities during retrieval attempts between the frontal lobe system – important for autonoetic consciousness, search initiation and monitoring, and the temporo-amygdalar system – important for emotional processing (Reinhold and Markowitsch, 2007, 2009). This memory impairment is opined to be triggered by adverse life conditions, usually of a recurrent nature and with onset in childhood or early adulthood; it is modulated by several factors, such as genes, personality characteristics, early parent-child attachment style, medical and psychiatric comorbidities, familial, ecological, and socio-cultural environment and epigenetic factors. Although some authors pointed to a direct relationship between the severity of exposure to trauma and incidence of dissociative amnesia (see the review of Maldonado and Spiegel, 2008), Markowitsch and co-workers described several patients who developed functional or dissociative amnesia after a seemingly objective minor stressor (Markowitsch, 2003; Fujiwara et al., 2008). This stressor was either psychological or physical (such as mild traumatic brain injury or mild physical injury) or a combination of the two. In most of these patients, a careful anamnesis revealed a history of recurrent stressful experiences with onset in early life, suggesting a mechanism of kindling sensitization or an incubation effect. In a substantial number of cases, functional amnesia co-occurred with a background of immigration and was attributed to a high allostatic load in this population. “‘Allostatic load’ refers to the price body pays for being forced to adapt to adverse psychosocial or physical situations, and it represents either the presence of too much stress or the inefficient operation of the stress hormone system” (McEwen, 2000, pp. 110–111). Psychological stress is however not considered to be sufficient for the development of functional amnesic condition. This was already implied by Janet, who reportedly suggested that the impact of trauma on a particular individual may dependent on a variety of factors (such as the personality features, prior experiences and the intensity, duration, and recurrence of the trauma) and might not become obvious immediately, but after a certain latency period (van der Kolk and van der Hart, 1989). Along the same line, Markowitsch and his colleagues have proposed that traumatic experiences interact in a time sensitive manner with genetic dispositions (with protective or deleterious effect; Becker-Blease et al., 2004) and other environmental factors (with buffering or exacerbating effects; Staniloiu et al., 2010b). In order to better causally model the emergence of functional amnesia or other psychopathological conditions in migrants, Markowitsch and co-workers supported proposals for a broadening of the conceptual understanding of the environment to encompass elements of the perinatal environment, personal life style habits and family, socio-cultural, and ecological environment (Hinton et al., 2008; Charney, 2012).

Changes in environment (especially these occurring in early life) were hypothesized to lead to genomic adaptations with consequences for stress hormonal response pattern, brain synaptic plasticity, and processing of incoming information (Markowitsch, 2003; cf. also Fries et al., 2005; McGowan et al., 2009). Several key brain structures for autobiographical memory and emotional processing have been identified as being sensitive to the consequences of exposure to stressful experiences (such as amygdala and hippocampal formation, prefrontal cortex, and specific white matter tracts). The effects of stress on the above mentioned brain structures depend on their vulnerability, the magnitude of the neurotoxic (glucocorticoid) cascade, and the stage of development or declining of the respective structures (Lupien et al., 2009). This may partly explain why the same type of traumatic experiences results in different brain morphological or functional changes and a multifarious, Iliad-like psychopathology (Briquet, 1859/1999; de Kloet and Rinne, 2007).

The hypothesis that increased glucocorticoid levels play a significant role in the mnestic blockade has received empirical support from several studies (for a review, see de Kloet and Rinne, 2007). The right hemispheric synchronization abnormality during retrieval attempts in functional or dissociative amnesia was suggested by a number of functional neuroimaging studies. In a study in which glucose-PET data obtained at rest from 14 patients with dissociative amnesia and severe retrograde episodic-autobiographical memory impairments were analyzed, it was found that the right temporo-frontal region was hypometabolic in a significant number of patients, with a significant reduction in the right inferolateral prefrontal cortex (Brand et al., 2009). A reinforcement of these results came in the same year from the work of Tramoni et al. (2009), who in a patient with functional amnesia performed magnetization transfer ratio measurement and MR spectroscopic imaging and found evidence of significant metabolic and subtle structural changes within the white matter of the right prefrontal region.

#### The psychological stress model: stress and episodic-autobiographical memory consolidating defect

As mentioned above cases of functional anterograde amnesia in the absence of retrograde episodic-autobiographical memory impairments have rarely been reported (Staniloiu et al., 2010a). As a consequence, neuroimaging data on these cases are sparse and inconclusive (Smith et al., 2010). We have encountered several cases of functional anterograde amnesia (Markowitsch et al., 1999; Staniloiu et al., 2010a). We conjecture that a stress hormone mediated consolidating deficit may partly account for the observed memory impairments. The stressful experience may lead to a consolidating defect by directly impacting on the function or structure of brain regions involved in the consolidation of memory and/or by afflicting the wake-sleep cycle (Peigneux et al., 2010; Lynn et al., 2012).

#### The executive deficit model in functional amnesia

Kopelman (2000) proposed a different model for the psychogenic retrograde amnesia that is however not mutually exclusive with the one advanced by Markowitsch. Kopelman posited that the inability to retrieve personal events in psychogenic amnesia results from an increase in the activity of inhibitory regions of the prefrontal cortex coupled with a subsequent decrease in the activity of the hippocampus, similar to the one that occurs in motivated forgetting. In line with Kopelman’s suggestions, Fujiwara and Markowitsch (2003) argued that the executive control – or supervisory attentional system – is engaged in holding unwanted or stressful memories out of self-awareness or autonoetic consciousness. This may result in an overload of the executive system and may reduce frontal capacities necessary for successful retrieval of other non-traumatic or non-stressful personal memories in psychogenic amnesia. In agreement with this model are findings suggesting that executive functions may co-vary with successful retrieval of autobiographical memory (Brand et al., 2009). In the study of Brand et al. (2009), executive dysfunctions were found in four patients. These patients showed more pronounced retrograde memory impairments than those with normal performance on executive function tests. It is worthwhile mentioning that both Kopelman’s emphasis on executive functions and Fujiwara and Markowitsch’s elaboration of the supervisory attentional system may have been anticipated by Janet (1907). Janet alluded to problems with executive functions in hysteria. Furthermore, he linked memory problems in psychogenic amnesia to attentional deficits. He wrote: “In fact, this *incapacity of attention* brings with it, as a consequence, *the absence of memory*” (p. 314).

#### Motivated forgetting

The possibility of the existence of an overlap in functional amnesia between “true” amnesia and simulation has been acknowledged since long (Lennox, 1943; Barbarotto et al., 1996; Jenkins et al., 2009). In a subset of patients with functional amnesia there may be an initial preponderance of conscious feigning or exaggeration of symptoms coupled with conscious mechanisms that draw on motivated forgetting or cognitive avoidance (Anderson and Green, 2001; Ortega et al., 2012). With repetition and passage of time, the conscious use of these mechanisms may “fade” into unconscious or semiconscious behaviors (Carter, 1853; Erdelyi, 2006) which act in the favor of preserving self-deception (Smith et al., 2010). This shift may get translated at the brain level in alterations of functions and/or structures (Ganis et al., 2003; Reinhold et al., 2006). For example, a study that investigated the neural correlates of deception showed right prefrontal activation associated with rehearsed lies that were part of a coherent story (Ganis et al., 2003). Although the latter results could be interpreted in various ways, one hypothesis is that through rehearsal, initiated by motivated agendas, self-related deceptive material may become familiar to our “narrative” self to the point that the deceptive material is not only part of a story we tell others, but also part of a story we tell to ourselves and we may live by Markowitsch and Staniloiu (2011a). New research data show that mechanisms that capitalize on memory suppression become more difficult with age (Anderson et al., 2011). This may partly explain why most cases of functional amnesia are diagnosed in the young age (Coons and Milstein, 1992; Reinhold and Markowitsch, 2007; Maldonado and Spiegel, 2008). Incidentally, negative correlations were found between age and dissociation scale scores (Putnam, 1997).

#### The impairment in emotional colorization and first person autonoetic connection

Markowitsch and co-workers (Markowitsch, 1995a,b, 1996; Markowitsch et al., 1997; Welzer and Markowitsch, 2005) as well as other researchers (Levine et al., 2009) observed that although a substantial number of patients with severe retrograde amnesic conditions might still be able to acquire new memories for long-term storage, these anterograde memories might lack the accompanying first person autonoetic emotional engagement and connection (Fujiwara and Markowitsch, 2006; Eich et al., 2009; Markowitsch and Staniloiu, 2011a). The above changes in anterograde conscious mnemonic processing could however escape capturing by standard anterograde memory tests and might subsequently require more sophisticated and refined neuropsychological testing coupled with functional neuroimaging investigations (Levine et al., 1998, 2009).

#### The binding deficiency model

Deficits in binding and reassembling details of the personal past events may partly account for retrograde episodic-autobiographical memory impairments (Fujiwara and Markowitsch, 2006; Rosenbaum et al., 2009), such as in cases of functional amnesia with malfunction of hippocampal formation (Markowitsch et al., 1998, 2000a).

#### The fantasy proneness model or the cognitive failures model (errors by commission)

The debate surrounding fantasy versus false memory versus true memory of trauma has persisted since the Freud’s time (for a review, see Mitchell and Black, 1995) and has occasionally been marked by incisive and unilateral positions, leading to break ups between various schools of thought (for a review, see Mitchell and Black, 1995). Adherents to the fantasy model have argued that the reports of traumatic memories in dissociative disorders are largely errors of commission, due to fantasy proneness, suggestibility, or cognitive failures (e.g., executive dysfunctions; Dalenberg et al., 2012). Some authors described a heightened risk of false memories (including confabulations) in patients predisposed to dissociation (Lynn et al., 2012). A systematic and rigorous evaluation of the susceptibility for false memory syndrome (confabulations, intrusions, false memory recognition) in patients with functional or dissociative amnesia however is missing (Fujiwara, 2004; Kritchevsky et al., 2004; Borsutzky et al., 2008, 2010; Dalenberg et al., 2012).

Hypnotizability traits have been postulated to be associated with a higher tendency for developing dissociative symptoms (Maldonado and Spiegel, 2008), though studies investigating the relationship between trait hypnotizability and risk for developing various dissociative (conversion) disorders yielded mixed results (Bell et al., 2011). Albeit hypnosis may facilitate access to dissociated memories in certain psychogenic amnesic conditions, there is some evidence suggesting a higher likeliness of confident errors accompanying hypnosis recall (Maldonado and Spiegel, 2008).

In a recent review of fantasy proneness model, Dalenberg et al. (2012, p. 1) concluded that “dissociation remains related to trauma when fantasy proneness is controlled.” The authors furthermore stated that they “find little support that dissociation–trauma relationship is due to fantasy proneness or confabulated memories of trauma” (p. 1).

#### The loss of information model

Both normal (Spiegel et al., 2011) and pathological forgetting (in the sense of loss of information; Huppert and Piercy, 1979) may occur in patients with functional amnesia. In patients with functional retrograde amnesia, who recover most of the conscious memories of the personal past, but continue to experience difficulties with recovering information from the period just prior to the traumatic incident, loss of information may be a possible explanation. This loss of information (Ribot, 1882) could be attributed to the fact that those particular memories were not fully “organized” (Burnham, 1903; Markowitsch, 1992) or consolidated (Haist et al., 2001) at the time of the incident. Loss of information may also occur in patients with functional amnesia, who suffer global intellectual deterioration over time (Markowitsch, 1992; Ladowsky-Brooks and Fischer, 2003). Porter and Landfield (1998) underlined the connection between the stress-related cognitive decline, the potential contribution of glucocorticoids to accelerated aging, the fronto-temporal dysfunction, and the later development of dementia. Since then a number of studies pointed to the relationship between psychological stress and dementia (e.g., Yaffe et al., 2010). There are however no systematic studies looking at the relationship between functional amnesia and dementia.

#### Socio-cognitive models of functional amnesia

The episodic-autographical memory is a superior neurocognitive ability, which is likely affected by genes, environment – including the socio-cultural milieu – and their interplay (Markowitsch and Staniloiu, 2012a). Social learning and expectancies, interaction with third parties (therapist cueing, health insurance policies), media influences, culturally molded sensation schemas, and explanatory models of illness may shape, perpetuate, or magnify the symptomatology in functional amnesia (Hinton et al., 2008). Linden et al. (2012), analyzing the manifestations of trauma during World War I, tried to raise the awareness that psychological manifestations of trauma may change their nature over time. “Clinical phenomenology is likely to have been a product of the type of trauma, the medical models available at the time and other cultural factors, but their exact interplay is still a matter for research” (p. 262).

In cases of functional amnesia occurring after a mild traumatic brain injury, the so coined diagnosis threat itself may influence neuropsychological test performance and symptom reporting. The terminology “diagnosis threat” derives from the concept of stereotype threat (Steele and Aronson, 1995), when a group member shows dismal performance on a task opined to be difficult for the group (Ozen and Fernandes, 2011).

### Differential diagnosis of functional amnesia

Functional amnesia has to be differentiated from amnesic disorders preponderantly etiologically linked to a general medical condition, amnesic disorders for substances, or gene-memory impairments accompanying various psychiatric conditions (for a review, see Markowitsch and Staniloiu, 2012a). Furthermore true amnesia has to be distinguished from feigned amnesia, which is not always an easy task. As Spiegel et al. (2011, p. 835) recently emphasized “in cases where the DA (dissociative amnesia) involves distress over current life conflicts or indiscretions, there may be a mixture of dissociative and factitious/malingered elements, making for a difficult differential diagnosis.”

### Case report – the search for modeling “forgetting” in functional amnesia and its discontents

“Many patients and their symptoms radiate away from the prototype,” but that does not mean that they are not true functional amnesic cases, “anymore than the oddities of an ostrich make it any the less a bird.” (Hacking, 1995, p. 32)

Mrs. X is a 47-year-old right-handed married woman, who shortly after turning age 36 years incurred a mild traumatic brain injury due to a car accident. Since the accident, Mrs. X has developed numerous somatic and psychological symptoms that have resulted in significant global functional impairment and inability to work. Incidentally, the accident incurred by Mrs. X occurred approximately a year after Mrs. X’s husband was diagnosed with an illness that interfered with his own working ability. At the time of Mrs. X’s referral for the present neuropsychological assessment (that took place 11 and 12 years after her accident), Mrs. X had provisional diagnoses of postconcussive syndrome or commotion cerebri and was receiving treatment with anti-migraine medication (non-steroidal anti-inflammatories) and ergotherapy. For the purpose of the assessment, a clinical interview was conducted with Mrs. X, her husband and the aid of a mother tongue (Hispanic language) interpreter. Copies of medical records, which Mrs. X made available to the present investigators, were also reviewed. Informed written consent was obtained for the participation in the study and publication of the report. The study adhered to the declaration of Helsinki.

A consistent accident history was provided by the patient’s husband and copies of reports from various health professionals involved in her care or assessment. Mrs. X was a pedestrian at the time of the accident. While she was lawfully crossing the street along the pedestrian line, she was hit by a car that was reportedly driving at low speed. The accident was witnessed by Mrs. X’s sons, who were accompanying her.

After the accident Mrs. X was immediately taken to the emergency room of a neighboring hospital, where she spent 3 days as an inpatient. In the hospital she was noted to be lightly dazed and she scored 14 on the Glasgow Coma Scale. She reportedly presented with both anterograde and retrograde amnesia for the events from the time period surrounding the accident. The pupils were symmetrically isochoric and normally reactive to light. There were no signs of focal neurological abnormalities. On physical exam, Mrs. X was noted to have a right frontal superficial laceration with the longest diameter of 3 cm and a swollen and painful left hand. A low grade fracture of the left scaphoid was diagnosed and Mrs. X received a left forearm volar splint, which she apparently wore for 3 months. Given the observed persistence (but no further deterioration) of the slight obnubilation a head computer tomography (CT) was performed. The head CT was negative for signs of skull fracture, intracerebral bleeding, or other pathological changes. The lacerated wound was treated locally. The patient was discharged home after 3 days, with a diagnosis of cerebral commotion and left scaphoid fracture.

There was no formal medical documentation of loss of consciousness at the time of the accident. The patient however reported later that she was told by the family members that she had lost consciousness at the time of the accident. The husband stated that he went to see Mrs. X in the hospital immediately after her admission, but she did not remark his presence in the first 24 h. Initially, she reportedly appeared unable to say anything. Later she asked him who he was. The husband also reported that several hours later, Mrs. X could only say her date of birth, but seemed unable to give any information about her children or family and she did not seem to know how she had gotten in the hospital. Over the month following the accident, Mrs. X’s clinical picture pronouncedly and gradually worsened and has pretty much remained so since then. She presented with difficulties focusing attention and concentrating, progressive memory impairments of both retrograde and anterograde nature, word finding difficulties, markedly decreased ability to communicate in the second (Roman) language (Calabrese et al., 2001), difficulties with calculations, massive psycho-motor retardation, apathy, and significant impairment of functioning. She reportedly showed growing difficulties with independently managing the household chores, which she had done before, with the consequence that they had to either be undertaken by her husband or performed by her under his close supervision. She herself complained of forgetfulness first approximately 3 weeks after the accident, though her insight into the extent of her memory difficulties seemed to be only partial. In addition, she did not seem to exhibit a high degree of concern about her neurocognitive difficulties. During assessments with various examiners or treating health professionals, she usually volunteered very little information spontaneously. Her complaints mainly consisted of somatic symptoms such as headaches, preponderantly located on the right side, neck pain, dizziness (when taking the stairs down or changing position from squatting to standing), left shoulder pain, double vision, fatigue, sensitivity to noise, and disturbed sleep. According to the husband, shortly after the accident Mrs. X experienced middle insomnia and easy startle responses to traffic noises, but no nightmares. Most recently, she showed increased need for sleep, although she described her sleep as being non-refreshing. She reported difficulties with remembering what tasks she was supposed to do in the house, stating that she would forget them after a few minutes. She complained of discomfort with being alone in her house and also expressed no desire to leave the house alone. When questioned about particular fears, she denied. However, her husband reported that Mrs. X would often seem distressed when crossing the street and would subsequently cling to him. Mrs. X’s husband portrayed Mrs. X as having been an energetic, daring woman prior to her accident. He described a marked change in her personality and behavior after the accident and perceived her as being apathetic and disinterested in most things she used to enjoy before. He remarked that Mrs. X still derived pleasure from being with him and their two sons. In addition he noted that she seemed to enjoy spending time and playing with small children (offspring of relatives or acquaintances). He stated that Mrs. X appeared to have difficulties with correctly appreciating distances between objects as well as judging the feelings of others. According to the husband, during a trip back to her hometown, Mrs. X had marked difficulties recognizing her relatives and showed signs of distress. Mrs. X’s eating preferences appeared to have changed since the accident and she reported an increased consumption of sweets.

In terms of medical and laboratory work up, a repeated neurological exam of Mrs. X performed 1 year after the accident was significant for anosmia (bilaterally), decreased ability of standing on the left leg and a tendency to deviate on the right side during the Unterberger test.

An MRI of her brain performed 2.5 years after the accident was described as unremarkable; a regular EEG undertaken 1.5 years after accident showed rapid alpha-rhythm with increased beta-activity. There was no evidence of seizure discharges. The EEG was formally read as yielding no pathological findings.

With respect to treatments, ergotherapy treatment was partly helpful with respect to fine finger movement and coordination. According to Mrs. X and her husband, Mrs. X had no history of alcohol or illicit substance use and no psychiatric or medical illness prior to the car accident (information that was consistent with previous documentation on file). The family history revealed that both of her sons developed difficulties with depressed mood after the onset of Mrs. X’s difficulties with memory and other cognitive functions.

The developmental history revealed that Mrs. X was born and raised in a small town in a family with five children. According to information obtained from the husband, Mrs. X was apparently the product of a normal pregnancy and delivery and achieved normal developmental milestones. Her childhood was uneventful. She completed 8 years of schooling.

Mrs. X came to a central European country at age 29, where she obtained a legal working permit. In this country, she successfully worked on a part time basis as a cleaner until her accident, without any conflict or significant absence. At the time of the present neuropsychological assessment, Mrs. X was deemed unable to work and she was receiving disability benefits. Mrs. X denied any history of developmental abuse, assault, torture, or political persecution. She also denied any marital conflict and reported a good relationship with her husband and children.

## Materials and Methods

### Neuropsychological assessment

The following tests were administered (or attempted to be administered):

*Tests for the estimation of intelligence and overall cognitive status* Abbreviated Wechsler Adult Intelligence Test-Revised (Block test and Picture Completion test; Dahl, 1972); DemTect (Calabrese and Kessler, 2000).
*Tests for the evaluation of attention, concentration, and processing speed* Trail Making Test A and B (TMT-A + TMT-B; Lezak, 1995); Attention Index of the German version of the Wechsler Memory Scale-R (WMS-R; Härting et al., 2000). Short-term memory and working memory (WMS-R; digit span and block span forward and backward).
*Tests for the evaluation of constructional functions* [Copy administration of the Rey–Osterrieth Figure Test (Osterrieth, 1944; Lezak, 1995)].
*Tests fort the evaluation of the verbal and non-verbal explicit anterograde long-term memory* Wechsler Memory Scale-R; Rey–Osterrieth Figure; Copy trial followed by delayed recall after 30 min (Lezak, 1995).
*Tests for the evaluation of explicit retrograde memory* Semantic knowledge (Schmidtke and Vollmer-Schmolck, 1999); Bielefeld Autobiographical Memory Interview BAGI; Fast et al., 2012.
*Test for evaluation of prospective memory* Recalling to perform an intended future action, in particular to ask at the ending of the testing for a personal object that the examiner had borrowed from the patient and hid it in the examining room, in response to a pre-specified time-based cue, namely the ending of the testing. Tests for the assessment of executive functions, including problem solving, cognitive flexibility Trail Making Test-B (Lezak, 1995); Tower of Hanoi (Lezak, 1995).
*Tests of verbal fluency* Controlled Oral Word Association Test [COW]; Supermarket task (word production); Boston-Naming Test (Lezak, 1995).
*Tests for evaluation of malingering tendencies* Test of Memory Malingering (TOMM; Tombaugh, 1996; Teichner and Wagner, 2004); Rey 15-item-test.
*Tests for evaluation of emotional processing* Florida Affect Battery, Subtests 1–3 (Facial Discrimination; Facial Affect Discrimination; Facial Affect Naming; Bowers et al., 1991; Emotional Pictures Test; von Cramon et al., 1993).
*Tests for mood screening* Beck Depression Interview (BDI; Beck et al., 1995).

As the patient was difficult to test, additional information was obtained in interviews with her. Prior to starting the testing process, Mrs. X. was asked a series of general interview questions, to assess for problems with awareness and orientation. She was unable to correctly identify her age, date of birth, date at the time of the assessment, location of assessment, or home address. When queried about her current mood, she described it as “tired.” Mrs. X’s husband confirmed her current limitations in functioning and stated that he has had to take over many of the tasks that she assumed responsibility for previously (e.g., organizing finances, cooking). She reported that she has neither “wishes” nor the “desire” for anything, apart from “sleep,” watching soap operas and spending time with her husband and sons or playing with small children.

## Results

### General behavioral observations

Mrs. X came to the assessment accompanied by her husband, sat near him and often tried to turn to him for the right answers or reassurance during the interview. She looked younger than her documented age and her behavior appeared to be childish. She volunteered spontaneously little information about herself. When queried, she often answered with very short sentences or used single words or two – word utterances or short telegraphic utterances. At times, instead of giving a verbal answer she just pointed to an object representing the answer with her index finger. She at times seemed to play with language, exaggerating the articulation of the syllables of certain words (such as her given name). When she spoke with longer sentences, her speech was fluent and there was no evidence of paraphasias. Although, overall, her comprehension of the mother tongue was good, Mrs. X at times turned to her husband for the explanation of a word. For instance, she had claimed not to understand the meaning of a name designating a well-known European country and, similarly to another patient with functional amnesia described in the literature by De Renzu et al. (1997), she then seemed puzzled to find out that it was a country. She had marked difficulties with naming the days of the week or the months of the year in temporal order and used her fingers to count them (De Renzu et al., 1997).

When asked about her age, Mrs. X adamantly stated that she was 35-year-old (misjudging her age by approximately 12 years). She ignored the statements of her husband, who tried to contradict her. The husband attributed her misjudgment of age to her fear of becoming old. Mrs. X was not able to provide her date of birth, however. She did not know the date, day of the week or year. When asked about the season, she responded “cold” (the interview indeed took place in the winter). She then inquired “Why am I here?” When asked, how old her children were, she responded “big,” implying that they were grown up. When asked, whether the children were still at home, she said “school” – implying that they still were going to school. She did not know the first names of her children, but said that the younger one was taller than the older.

Mrs. X could not explain the discrepancy between her self-reported age and having grown up children, which was pointed out to her by her husband. She however did not seem concerned about this discrepancy and stated that she knew that they were her children because they call her “mother.” When Mrs. X was asked about ever feeling like someone who failed, she seemed to be offended by this question (which was part of the BDI). She replied that she felt an accomplished woman because she had two sons and a loving husband. While saying this, she pointed to her husband with her index finger, smiling childishly.

When asked about her childhood, Mrs. X provided very little information. She did not know anything about her school time. She just said that she lived with her mother in a house. When she was asked whether she had brothers or sisters, she first did not respond. When the examiner reformulated the question in a forced choice format asking her if she had one or two siblings, Mrs. X responded “three.” She stated that one was a brother. For one sister she gave her first name. (Mrs. X’s husband later stated that Mrs. X in fact had five siblings).

When asked where she was born, Mrs. X first said “no,” then she correctly named her country of origin and added that it was “warmer” there. When she was queried about her activities during the day, she first did not respond. When she was asked explicitly: “What do you do in morning, what during lunch time, what in the afternoon?” she said “morning-problem, afternoon-better after rest.”

When the interviewer inquired whether she watched TV, Mrs. X responded: “nice soap operas.” When she was queried about her ability to recall the content of the watched soap operas, she responded she would know the persons: “names no; good person, bad person, yes.” Her husband confirmed that Mrs. X indeed spent time watching TV on a daily basis, except for Sundays. He added that initially after the accident Mrs. X had marked difficulties with distinguishing the TV reality from the true outer world reality. She also had complained initially of difficulties with following a movie.

Sometimes Mrs. X would reportedly go for shopping in a small store, but she avoided bigger stores, for reasons she was not able to verbalize. She would leave the house infrequently and when she did so she was most of the time accompanied by her husband. She reportedly had re-learned how to use the washing machine, but would not do the laundry.

During the initial interview and the ensuing testing, Mrs. X often played with her fingers and covered both hands with the sleeves of her jacket. She at times shook her leg and exhibited grimacing movements of her lips. She denied feeling anxious, however. She frequently complained of feeling tired, rubbed her eyes with her hands, and yawned. On a couple of occasions, she asked if the testing was over so she could go home and sleep.

All the testing instructions were given to her in the mother tongue, though it is unclear if Mrs. X always fully comprehended them. Mrs. X tried to give an answer for each test item, but her level of engagement and effort was inconsistent. When she had to attend to tasks involving potentially gratifying stimuli, her level of engagement and alertness seemed to increase, however.

Overall, observations of Mr. X’s approach to tasks suggested that she might not have put forth a concerted and consistent effort. This may be congruent with her exceptionally poor performance on nearly all tasks, including measures of memory malingering. Consequently, some test results might not be a valid indicator of her cognitive abilities. However, a qualitative account of her responses and behavior during the testing process is described below.

### Intelligence and overall cognitive status

#### Abbreviated Wechsler intelligence test

From the intelligence test subtests Mrs. X was able to construct patterns as presented on a model, but needed a long time (140 s for the simplest patterns of the Block test). In the Picture Comprehension subtest she only detected missing parts, after the examiner had pointed out in what area the missing part was located or what it might have consisted of. She only pointed with her finger, but did not name certain missing parts, such as the leg of a crab.

#### DemTect

The patient was not able to engage in the first part of the test, consisting of a word list. Subsequently, no other subtests were administered.

### Tests for the evaluation of attention, concentration, and processing speed

#### Trail making test A and B

Mrs. X managed to complete the practice samples of the TMT-A + B. She however completed the testing part of the TMT-A with significant difficulty, requiring constant correction and redirection after she had made several errors. She was unsuccessful with completing the testing part of the TMT-B.

#### Attention index of the German version of the Wechsler memory scale-R

The patient did not engage in these tests.

#### Copy administration of the Rey–Osterrieth figure test

Mrs. X reached 28 points (below the 10th percentile of matched controls).

### Tests for the evaluation of the verbal and non-verbal explicit anterograde long-term memory

#### WMS-R (logical memory test)

The patient had significant difficulties with answering questions pertaining to personal and current information and orientation. She stated that her age was 35 years (roughly the age when the accident occurred) instead of 47 years. On Mental Control tests, she could not say the alphabet; she only said “D,” when the examiner started with “ABC.” Mrs. X only could repeat a few single digits. Only one of the two stories of the Logical Memory Test was administered and on this test Mrs. X gained 4 out of 25 points at immediate recall. After 30 min Mrs. X did not recall anything. Since Mrs. X’s performance was already deemed severely impaired on the above conditions, other subtests were not administered.

#### Rey–Osterrieth figure 30 min recall

Mrs. X performed very poorly (see Table 1).

**Table 1:** **Summary of test results**.

	Raw score	Comment
**ATTENION, CONCENTRATION**		
Trail making test A + B	A: 123 s, 1 error; B: not applicable	Far below average
**INTELLIGENCE**		
Abbreviated Wechsler intelligence test	Only half of the tests solved	Far below average
**ANTEROGRADE MEMORY PERFORMANCE**		
Rey–Osterrieth figure, copy	28 Points	Below average
Rey–Osterrieth figure, redrawing after 30 min	5 Points	Far below average
**EMOTIONAL PICTURE RECOGNITION**		
Recognition of emotional pictures	33 Errors (out of 80 stimuli)	Far below average
**REMOTE MEMORY PERFORMANCE**		
Bielefeld autobiographical Memory	No answers	No evidence for recall of autobiographical
Interview		episodes
**PROSPECTIVE MEMORY**		
Remembering of having given an item to the tester	Remembrance only after several reminders and hints	Below average
**PROBLEM SOLVING ABILITY, COGNITIVE FLEXIBILITY**		
Tower of Hanoi (three-disk-version)	Only after the third demonstration she succeeded in solving it	Far below average
Controlled oral word test	No adequate response	Far below average
Supermarket task	4 Responses	Far below average
Boston-test, naming	5 out of 60 correct; frequently only descriptions	Far below average
**EMOTIONS, FEELINGS, THEORY OF MIND-ABILITIES**		
Florida affect battery
Facial discrimination (subtest 1)	75% Correct	Far below average
Facial affect discrimination (subtest 2)	75% Correct	Far below average
Facial affect naming (subtest 3)	55% Correct	Far below average
Emotional picture recognition	33 Errors (out of 80 stimuli)	Far below average
**TENDENCIES FOR MALINGERING**		
15-Item-test	Nothing correctly reproduced	Malingering
		Questionable
TOMM, first trial	26/50	Malingering
		Questionable
TOMM, second trial	15/50	Malingering
		Questionable
TOMM, recognition	23/50	Malingering
		Questionable
Beck depression inventory	Answers to only part of the questions	No tendency for depression

### Tests for the evaluation of explicit retrograde memory

#### Bielefeld autobiographical memory interview

Mrs. X could not provide any memory of a past personal event that would satisfy criteria for having an episodic-autobiographical quality (Tulving, 2005).

#### Semantic general knowledge

Mrs. X had marked difficulties with answering questions concerning famous events, historical names, and geographical names.

### Test for evaluation of prospective memory

The examiner requested a personal object (the wedding ring) from the examinee at the beginning of the testing session. The examinee was instructed to ask the examiner for the return of her personal object at a specific point in time in the future. The patient did not ask for it spontaneously in response to the pre-specified time-related cue. Only after being given several verbal cues by the examiner, the examinee recalled that her wedding ring had been taken by the examiner and consequently asked for it being returned to her.

### Test for evaluation of executive functions

#### Trail making test-B

The patient performed very poorly on this task.

#### Tower of Hanoi

The patient was unable to successfully complete the task. The patient was unable to follow the instructions. Only after the examiner demonstrated (simplest) three-disk-version task twice, Mrs. X was able to perform it as well, but she still needed redirection and correction in order to prevent her from engaging in unacceptable movements. She therefore appeared to be unable to keep track of instructions even in simple, play-like situations.

### Tests of verbal fluency

#### Controlled oral word association test

She did not produce any word in response to a given phonemic cue (letter).

#### Supermarket task (semantic cue verbal fluency task)

Mrs. X just named “bread, fruits, juice, chocolate” (items that she in fact reportedly enjoyed eating).

#### Boston-naming test

She only could name very simple objects. Frequently she either gave perceptual descriptions or used terms that shared perceptual similarities: Instead of “mushroom” she said “lamp,” pointing to a table lamp close to her which had a mushroom-like shape. Instead of “cactus” she said “plant,” instead of “pencil” “something to write,” instead of “comb” “something for the hair,” instead of “toothbrush” “something for the teeth,” instead of “rhinoceros” “something like a cow,” instead of “unicorn” “horse.”

### Tests for evaluation of malingering tendencies

#### Rey 15-item-test

She was unable to reproduce the 15-items; she just wrote for the first line (with numbers 1, 2, 3) 1, 3, 5, 4.

#### Test of memory malingering

Mrs. X was given the TOMM in order to help determine whether she has bona fide memory impairments. Mrs. X scored extremely low on both recall and retention trials of this forced choice test. In the first trial she produced 26 out of 50 items correct, in the second, 15 out of 50, and in the recognition trial she answered 23 out of 50 correctly. This means that she performed in two trials nearly exactly at chance level and in one even considerably below chance level (i.e., below the 95% confidence interval for chance performance). When a non-demented examinee achieves a score below 18/50 (i.e., the lowest score that one can achieve within the 95% confidence interval for chance performance), it usually implies that the person knew some of the pictures were correct but intentionally picked the incorrect picture. In particular, performance on Trial 2 was traditionally found to be very high for non-malingers regardless of age. Mrs. X’s score on Trial 2 was lower (or worse) than her score on Trial 1 and below the chance level.

Test of memory malingering is sensitive to malingering and/or lack of effort. A low score on the TOMM has traditionally suggested that either the individual’s memory impairment on the test is false or exaggerated or that the low score is due to lack of effort. According to newer data, TOMM however appears sensitive to detect the severe cognitive dysfunction associated with dementia. Therefore TOMM might not be a useful measure of assessing test motivation for persons with severe intellectual global deficits, such as in dementia (Teichner and Wagner, 2004).

While we cannot fully rule out an exaggeration of memory impairment in the case of Mrs. X, we are of the opinion that her low performance on TOMM might be better accounted for other factors, given that her performance on all neuropsychological tasks was dismal (Kessler et al., 1997; Sollman and Berry, 2011). These factors consist of a global cognitive deterioration coupled with a lack of concerted and consistent effort. Mrs. X approached some of the tasks in a seemingly playful, childish manner and showed an increased interest and level of attention for potentially positive reinforcing stimuli, such as particular food items (“ice cream”), flowers. There is also a suspicion that Mrs. X may have not fully understood the instructions, in spite of that they were communicated to her by the interpreter in her mother tongue and that the examiner periodically queried her about her comprehension of instructions.

### Tests for evaluation of emotional processing

#### Florida affect battery, subtests 1–3

With scores of twice 75% (Facial Discrimination and Facial Affect discrimination) and 55% (Facial Affect Naming), Mrs. X performed far below average.

#### Emotional pictures test

Also in recognizing previously seen emotional pictures she situates herself with 33 errors (out of 80 stimuli) even below chance level. It might be speculated that she just looked for novelty and had acquired only some familiarity or subconscious knowledge of the previously perceived stimuli (see Figure 1 and description of memory systems in Markowitsch and Staniloiu, 2012a). Novelty is universally found in mammals and probably vertebrates in general (Wilson and Rolls, 1990; Tulving et al., 1996).

### Tests for psychiatric symptoms screening

#### Beck depression interview

Mrs. X did not give an answer for all the screening questions. She reported increased need for sleep, decreased libido, difficulties with attention and concentration, and fatigue. Based on the psychiatric interview that was carried out with the patient and the collateral information obtained from her husband, a full constellation of symptoms suggestive of a diagnosis of major depressive disorder could not be elicited. Furthermore, no evidence of symptoms suggestive of a bipolar disorder or psychotic disorder was found. Mrs. X’s clinical presentation and history also did not satisfy diagnosis criteria for a post-traumatic stress disorder.

## Discussion

Unfortunately, a complete profile of Mrs. X’s cognitive strengths and weaknesses could not be ascertained. She scored extremely low on all tests of cognitive functioning, with little discrimination between tasks. In part she might have not understood all the instructions. Nevertheless, she did not refuse to perform and always appeared to try to give some kind of answer (e.g., Boston-naming test). Her performance was gaged as being below her premorbid intellectual status as estimated on the basis of the collateral information. Mrs. X’s husband told that prior to the accident Mrs. X always did the tax bills and other paper work for the family, and she behaved like a normal wife and mother for husband and family.

Mrs. X’s performance was dismal regardless of the functional domain, nature of the stimuli, task difficulty, or response mode. She performed poorly on tests tapping on attention, concentration, intelligence, executive functions, verbal fluency, anterograde and retrograde memory, verbal and non-verbal memory, semantic as well as episodic-autobiographical memory, prospective memory, and emotional processing. Also in tests of malingering which were based on remembering of visual items, she performed extremely deviant from the norm. Though these scores might be indicative of deliberate simulation, we opted for several alternative interpretations in Mrs. X’s case, for the following reasons. First, Mrs. X showed signs of global and severe cognitive impairments. Second, Mrs. X’s performance suggested that she was not putting forth her best effort. Thirdly, we could not identify any external incentives which might account for malingering.

Although Mrs. X’s neuropsychological profile might raise the suspicion of a deliberate effort to feign cognitive impairment, we considered a diagnosis of malingering very unlikely (Vrij, 2000; Boone, 2007). With respect to Factitious Disorder, a key difference between the later and the malingering is considered to be the nature of motivation (Mayou et al., 2005). According to the diagnostic criteria for Factitious Disorder, the individual is motivated to assume the role of a sick person. We did not find sufficient evidence to suggest that Mrs. X was deliberately trying to assume the sick role; however, this possibility cannot be fully ruled out. As several authors have already underlined, it is often difficult to accurately assess “the motivating goals that accompany reports of illness” (Bass and Halligan, 2007). Furthermore, “deception” is not unique to Factitious Disorder; instead, “symptom exaggeration” has been reported in several conditions, such as mild head injury, depressive disorders, and dissociative disorders (Krahn et al., 2008).

Based on the neuropsychological findings and the collateral information, we opined that Mrs. X’s clinical presentation could be viewed as the result of the complex interplay between psychological and biological factors and would be best captured by the construct of functional amnesia. Several authors remarked that a mixture in variable proportions of psychological and physical factors contribute to the emergence of functional disorders (Sollier, 1892; Kopelman, 2000).

Mrs. X’s symptoms had their onset after a traumatic brain injury with right localization (Schore, 2002; Markowitsch and Staniloiu, 2011a). This finding is congruent with other reports of functional amnesia occurring after a mild traumatic brain injury (Kopelman, 2000; Fujiwara, 2004; Serra et al., 2007; Maldonado and Spiegel, 2008). Although her traumatic brain injury was labeled as being mild based on the findings from Glasgow Coma Scale scores, neurological exam, and conventional structural imaging, a biological contribution of the traumatic brain injury to the emergence of her cognitive and affective symptoms cannot be ruled out. Multifocal diffuse axonal damage can result from mild traumatic brain injuries and escape capturing by conventional structural methods (Lipton et al., 2008, 2009). It can lead to executive dysfunctions as well as impairments of conscious mnemonic processing (“disconnection syndrome”; Markowitsch, 1988, 1992). Although conventional structural neuroimaging techniques typically do not show any significant structural brain changes in mild traumatic brain injuries, diffusion tensor imaging studies have provided evidence of temporally dynamic micro-structural changes of white matter after mild traumatic brain injury (Newcombe et al., 2012), which correlate with the patients’ performance on tasks, which tap on a variety of cognitive domains (Lipton et al., 2008, 2009).

In some cases of mild traumatic brain injury, the use of voxel based morphometry (Sehm et al., 2011) identified progressive volumetric changes in structures involved in mnemonic processing (such as the amygdala and hippocampal formation; Venugopal et al., 2012). Although Mrs. X did not present with gross neurological focal signs on physical examination, there were reports of subtle neurological abnormalities on the left side of the body. Of particular interest is the documentation of bilateral anosmia, which may explain Mrs. X’s changes in eating preferences. Anosmia may also affect the perception of the pheromones with negative consequences for intimate relationships (Reinhold and Markowitsch, 2009). Furthermore an association between changes in olfaction and memory impairments or alterations of the capacity for empathy has been described in literature (Wilson et al., 2007). Mrs. X presented with impairments in conscious mnemonic processing, social information processing (judging the feelings of others, face-emotion judgments) and capacity for intimacy.

As mentioned above, a number of patients with dissociative or functional amnesia described in the literature had the onset of their psychiatric symptoms after a traumatic brain injury (Kopelman, 2000; Staniloiu et al., 2010a,b). Similar to Mrs. X, several cases of the traumatic brain injuries were of mild severity (Miller et al., 1997) and therefore they were not considered to solely account for the onset of cognitive difficulties.

From a psychological perspective, the mild traumatic brain injury could be viewed in Mrs. X’s case both as psychological traumatic event and a legitimate escape from a life situation that may have appeared as inescapable (Kopelman et al., 1994). Although Mrs. X did not meet criteria for a diagnosis of post-traumatic stress disorder, she endorsed some symptoms of hyperarousal after the accident (easy startle to traffic noises, insomnia). The symptomatology later shifted to be dominated by hypersomnia, severe memory impairments, avoidant behavior and various somatic complaints. Mrs. X’s focus on somatic symptoms coupled with her scant verbalization of emotions suggest an impairment of her capacity for emotional processing in face of ongoing stressors. Apart from the traumatic brain injury, Mrs. X seemed to have encountered other stressful experiences prior to her accident, such as the one related to the disabling illness of her husband and the subsequent increase in her parental and care giving demands. These negative experiences may have been superimposed on a background of an already increased allostatic load, leading to a magnification of stress hormonal responses, with lasting consequences for her psychological well-being. As mentioned above, data from various sources point to an increased allostatic load in migrants which has been related to pre-migration, migration, and post-migration experiences (Staniloiu et al., 2010a). An association between alexithymia and dissociative and somatoform disorders and somatization has been corroborated by several studies. Connections between traumatic brain injury and alexithymia have also been reported (Koponen et al., 2005). Although a formal measure of alexithymia was not administered to Mrs. X, both her clinical presentation and her performance on other tests that tapped on capacity for emotional processing suggested deficits of emotional awareness and clarity. We speculate that these deficits may have pre-dated her mild traumatic brain injury, which in turn may have exacerbated them.

Apart from retrograde and anterograde amnesia, Mrs. X presented with symptoms suggestive of the Ganser syndrome (*vorbeireden* and *nichtwissen*; Magnin et al., 2012) as well as a conversion disorder, such as diplopia (Pommerenke et al., 2012). From a socio-cognitive perspective, we conjecture that social learning and expectancies may have played a role. As mentioned above, Mrs. X’s symptoms occurred approximately a year after her husband was diagnosed with an illness for which he received disability benefits, suggesting a possible unconsciously learned component to her behavior (Kopelman, 2000).

The onset of Mrs. X’s symptoms was sudden and the symptoms have persisted for more than 11 years. There are some indications that over the time her clinical presentation shifted, in the sense that she showed a lack of concern about her symptoms (*difference*; Janet, 1907) and future and signs of regression to pre-operational ways of thinking. She developed difficulties with judging the feelings of others (egocentrism). She seemed content to live in a noetic present, which preserved her juvenescence fantasies and perhaps her fears of death (Suddendorf et al., 2009).

Although not prototypical, Mrs. X’s case is in our opinion essential for understanding the functional amnesia. It lends support to the idea that functional amnesia is a heterogeneous, multifaceted condition, which extends beyond the episodic-autobiographical memory impairment (Markowitsch and Staniloiu, 2012b). It supports the idea that cases of functional amnesia with prominent anterograde memory impairments may be not as unique or exceptionally rare as portrayed in international nosologies (Spiegel et al., 2011). It provides an example of functional amnesia with a chronic course (Staniloiu and Markowitsch, 2010a). It links functional amnesia to an increased allostatic load, which results from both psychological and physical factors (similarly to transient global amnesia; Bartsch and Deuschl, 2010). It supports the incubation model of trauma described by Charcot or Janet (van der Kolk and van der Hart, 1989; Bogousslavsky, 2011) and the cumulative models of trauma (for a review see Mitchell and Black, 1995). Furthermore, it raises the awareness about a possibly increased frequency of dissociative amnesic conditions in migrants (During et al., 2011).

Additionally, the case points out to a gamut of possibly underlying “forgetting” mechanisms. Its chronic course suggests that loss of information may be one of these mechanisms, calling for larger prospective, longitudinal neuropsychological, and neuroimaging studies that can objectively quantify the changes in conscious mnemonic processing that may ensue in this condition over time.

## Conclusion

Sir Lewis (1963, p. 16) remarked the following: “The somatic pathology of mental disorder is well illuminated in those conditions in which the mental disturbance is invariably associated with abnormal structural or chemical changes of which it is a symptom; it is less clear when there are metabolic anomalies or fluctuations which are unspecific; and it is a very dark chamber, lit from time to time with tantalizing flashes, in the numerous ‘functional’ disorders.”

Although the strides made in the field of neuroscience have since then brought some light into the dark chamber of functional disorders, the “riddle” of functional amnesia (Lundholm, 1932) is far from being solved. Despite evidence pointing to its substantial prevalence, there is a dearth of data about the evidence-based treatment of functional amnesia (Markowitsch and Staniloiu, 2012a). This emphasizes the need for larger studies that should disentangle its neurobiological correlates and psychosocial underpinnings.

## Conflict of Interest Statement

The authors declare that the research was conducted in the absence of any commercial or financial relationships that could be construed as a potential conflict of interest.
